# Spasmolytic Effects of Aphanizomenon Flos Aquae (AFA) Extract on the Human Colon Contractility

**DOI:** 10.3390/nu13103445

**Published:** 2021-09-28

**Authors:** Antonella Amato, Simona Terzo, Pierenrico Marchesa, Angela Maffongelli, Martina Martorana, Stefano Scoglio, Flavia Mulè

**Affiliations:** 1 Department of Biological, Chemical and Pharmaceutical Sciences and Technologies (STEBICEF), University of Palermo, Viale delle Scienze, 90128 Palermo, Italy; simona.terzo01@unipa.it (S.T.); flavia.mule@unipa.it (F.M.); 2 U.O. Oncology Hospital, A.R.N.A.S. Ospedali Civico Di Cristina Benfratelli, Palermo, Via Carmelo Lazzaro, 4, 90127 Palermo, Italy; pmarchesa1@gmail.com (P.M.); angelamaffo@aol.com (A.M.); martina.martorana@hotmail.com (M.M.); 3 Nutritherapy Research Center, 61029 Urbino, Italy; stefanoscoglio@me.com

**Keywords:** AFA extract, Klamin^®^, human colon contractility, β-PEA, motility discomfort

## Abstract

The blue-green algae Aphanizomenon flos aquae (AFA), rich in beneficial nutrients, exerts various beneficial effects, acting in different organs including the gut. Klamin^®^ is an AFA extract particularly rich in β-PEA, a trace-amine considered a neuromodulator in the central nervous system. To date, it is not clear if β-PEA exerts a role in the enteric nervous system. The aims of the present study were to investigate the effects induced by Klamin^®^ on the human distal colon mechanical activity, to analyze the mechanism of action, and to verify a β-PEA involvement. The organ bath technique, RT-PCR, and immunohistochemistry (IHC) were used. Klamin^®^ reduced, in a concentration-dependent manner, the amplitude of the spontaneous contractions. EPPTB, a trace-amine receptor (TAAR1) antagonist, significantly antagonized the inhibitory effects of both Klamin^®^ and exogenous β-PEA, suggesting a trace-amine involvement in the Klamin^®^ effects. Accordingly, AphaMax^®^, an AFA extract containing lesser amount of β-PEA, failed to modify colon contractility. Moreover, the Klamin^®^ effects were abolished by tetrodotoxin, a neural blocker, but not by L-NAME, a nitric oxide-synthase inhibitor. On the contrary methysergide, a serotonin receptor antagonist, significantly antagonized the Klamin^®^ effects, as well as the contractility reduction induced by 5-HT. The RT-PCR analysis revealed TAAR1 gene expression in the colon and the IHC experiments showed that 5-HT-positive neurons are co-expressed with TAAR1 positive neurons. In conclusion, the results of this study suggest that Klamin^®^ exerts spasmolytic effects in human colon contractility through β-PEA, that, by activating neural TAAR1, induce serotonin release from serotoninergic neurons of the myenteric plexus.

## 1. Introduction

The blue-green algae Aphanizomenon flos aquae (AFA), also called Klamath algae, is a unicellular prokaryotic microorganism belonging to the Cyanobacteria phylum, growing in Upper Klamath Lake (Oregon, OR, USA) [1]. AFA is considered a “superfood” due to its particularly rich nutritional profile. In fact, it is an excellent source of proteins, polyunsaturated fatty acids, minerals, sterols, carotenoids, and phycocyanins [2], all substances which are well known for their antioxidant [3,4,5,6], anti-inflammatory [7], and anticancer [8] properties. The richness in healthy nutrients seems to be responsible for the numerous health-enhancing benefits derived from AFA consumption as a food supplement [9]. Interestingly, AFA contains significant amounts (8 mg/g) of β-phenylethylamine (PEA), a member of the trace-amine (TAs) family, a secondary amine system, which is present in relatively low amounts in the central nervous system (CNS) [10,11]. β-PEA is endogenously synthesized in the CNS as a by-product of catecholamine biosynthesis, and it is considered an important neuromodulator for dopamine, norepinephrine and serotonin (5-HT) release [12]. Accordingly, alterations in β-PEA synthesis have been documented in various neurological disorders including depression, affective disturbances [13] and Parkinson’s disease [14]. In contrast to the well-characterized action of β-PEA in the CNS, very little is known about β-PEA effects outside of the CNS. To date it is no clear if β-PEA could act as neuromodulator or neurotransmitter in the enteric nervous system (ENS), considered as the “second brain” because of high number of neurons and complexity in signalling pathways. In fact, there are few studies concerning the role of β-PEA in the ENS and, in any case, the results are controversial because β-PEA seems to be able to induce both contractile or relaxant response in the gut depending by the animal species or the segment of the digestive tract considered [15,16,17]. β-PEA exerts its actions by binding trace amine-associated receptor 1 (TAAR1), a family of G protein-coupled receptors [18,19] which are highly expressed in peripheral tissues, including mammalian gastrointestinal tract [20,21,22]. Nevertheless, the TAAR1 expression in human intestine remains to be confirmed.

It is likely that AFA extracts, once ingested as food supplements, exert effects also in the gastrointestinal tract, as it was proven in relation to the AFA-extract AphaMax^®^, rich in phycocyanins and phytochrome but poor in β-PEA, helpful for counteracting oxidative stress and inflammation [23]. Another AFA extract, Klamin^®^, concentrates β-PEA and has shown to be helpful for depression, anxiety, and mood disturbances [24,25,26].

The present research was undertaken to characterize the possible effects of exogenous administration of Klamin^®^ on human colon contractility. In particular, the mechanical responses of the distal colon circular smooth muscle, the mechanism of action underlying the observed effects, and possible β-PEA involvement were investigated using the organ bath technique.

## 2. Materials and Methods

### 2.1. Human Tissue Specimens and Preparation

Human colons were collected from 30 subjects (aged 61–87 years, 31% females) who underwent colectomy for sigmoid cancer at the Azienda di Rilievo Nazionale ad Alta Specializzazione (A.R.N.A.S.), Ospedali Civico Di Cristina Benfratelli-Palermo, Italy. The experimental protocol was approved by the Institutional Ethics Committee (HCP0617-June 2017; Comitato Etico CE Palermo 2 -ex D.A. n. 1360 del 16 July 2013) and all patients provided written informed consent before surgery. Samples consisted of normal colon collected from regions free of macroscopic evidence of cancer infiltration. The colonic specimens were immediately placed in preoxygenated Krebs solution to remove the mucosal layer, and then stored overnight at 4 °C. The other three samples were frozen and stored at −80 °C for subsequent TAAR1 expression analysis (RT-PCR) or fixed in cold 4% paraformaldehyde diluted in PBS for immunohistochemistry (*n* = 3).

### 2.2. Functional Studies

As previously reported [27], following the overnight storage, strips of 0.4 × 1 cm were cut parallel to the circular muscle and suspended in a four-channel organ bath in which each chamber was filled with 8 mL of Krebs solution with the following composition (mM): NaCl 119; KCl 4.5; MgSO_4_ 2.5; NaHCO_3_ 25; KH_2_PO_4_ 1.2; CaCl_2_ 2.5; glucose 11.1 and aerated with 5% CO_2_, 95% O_2_, pH 7.4 at 37 °C.

One end of each strip was tied to organ holders, while the other end was attached with a silk thread to an isometric force transducer (FORT25, Ugo Basile, Biological Research Apparatus, Comerio, VA, Italy). The mechanical activity was digitized on an analog-to-digital converter, visualized, recorded, and analyzed on a personal computer using the PowerLab/400 system (Ugo Basile Biological Research Apparatus, Comerio, VA, Italy). Tissue strips were initially stretched to 1 g and allowed to equilibrate for 60 min, during which they developed rhythmic phasic contractions. In each experiment, up to six strips from the same specimen were studied.

After the equilibration period, the effects induced by increasing dose of Klamin^®^ (5–35 mg/mL), AphaMax^®^ (5–35 mg/mL), or β-PEA (0.1–100 μM) on the spontaneous mechanical activity were examined to obtain dose-dependent response curves. The substances were added to the bath at increasing concentrations in volumes of 80 μL, and left in contact with the tissue for 5 min. The responses to Klamin^®^ and β-PEA were also tested in the presence of *N*-(3-Ethoxyphenyl)-4-(1-pyrrolidinyl)-3-(trifluoromethyl)benzamide (EPPTB) (50 μM), a TAAR1 antagonist. In order to examine the action mechanism, the Klamin^®^-induced effects were tested in the presence of tetrodotoxin (TTX; 1 μM), a voltage-dependent Na^+^-channel blocker, Nω-nitro-L-arginine methyl ester (L-NAME) (300 μM), an inhibitor of nitric oxide (NO) synthase, or methysergide (10 μM), a non-selective serotonin receptor antagonist. In another set of experiments the human colon responses to cumulative concentrations of 5-HT (0.1 μM–1 mM) were examined in the absence or in the presence of methysergide (10 μM). The concentrations of the drugs used were determined from the literature [28,29,30,31].

### 2.3. TAAR1 Expression Analysis

Gene expression: Total RNA from the colon sample was extracted using a PureLink RNA Mini Kit (Invitrogen, Carlsbad, CA, USA) according to manufacturer’s instructions and quantified by spectrophotometry. Hence, 1 mg of total RNA was reverse-transcribed using a High-Capacity c-DNA RT Kit (Applied Biosystems, Foster City, CA, USA). cDNA (5 μL; 30 ng total RNA equivalents per reaction) were subjected to RT-PCR amplification. The oligonucleotide primers were the following:

For: 5′-CTGTACAGTTTAATGGTGCTCATAATTCTGACC-3′; Rev 5′-AGCATAGTAGCGGTCAATGGAGATGAAAGAC-3′ from human TAAR1; For: 5′-CGGGATCCCCGCCCTAGGCACCAGGGT-3′; Rev: 5′-GGAATTCGGCTGGGGTGTTGAAGGTCTCAAA-3′, from human β-actin. Each PCR cycle employed a 3-min denaturing step at 95 °C followed by 40 cycles at 95 °C for 15 s, 58 °C for 30 s and 72 °C for 30 s and a final extension step of 7 min at 72 °C. The amplimers were separated on a 1% agarose gel containing 0.5 mg/mL ethidium bromide for visualization. The gel was scanned under UV light. Brain human cells was used as positive control [32].

Immunohistochemistry: Sections (5 μm thick) of paraffined-embedded colon were hydrated in a sequence of graded ethanol (from 96 to 70%) for 10 min each, washed in water and then PBS. The slides were incubated with Normal Horse Serum for 20 min and subsequently with purified polyclonal antibodies: Rabbit polyclonal against TAAR1 (6–15 µg/mL) (Abcam, Cambridge, UK) and Rabbit polyclonal against Tryptophan Hydroxylase (5 μg/mL) at room temperature overnight. This antibody is usually indicated to give a positive result in IHC for 5-HT in the human normal colon (https://www.abcam.com/tryptophan-hydroxylasetph-antibody-ab46757.html#description_references) (accessed on 26 August 2021). After washing, the samples were incubated with Biotinylated Universal anti-Mouse/Rabbit secondary antibody (Vector Laboratories, Inc. Burlingame, CA, USA) for 30 min. After being washed for 5 min in PBS, sections were then incubated with avidin/biotinylated horseradish peroxidase (HRP). Development was performed using the 3,3′-diaminobenzidine (DAB) Substrate Kit for Peroxidase (Vector Laboratories, Burlingame, CA, USA). Sections were then counterstained with hematoxylin and mounted routinely. The images were visualized using a Leica DM5000 upright microscope (Leica Microsystems, Heidelberg, Germany) at 20× magnification.

### 2.4. Drugs

The drugs used were the following: Klamin^®^ extract (kindly supplied by Nutrigea Research s.r.l., Borgo Maggiore, Republic of San Marino), TTX (Alomone Labs, Jerusalem, Israel), EPPTB (*N*-(3-Ethoxy-phenyl)-4-pyrrolidin-1-yl-3-trifluoromethyl-benzamide) (Hoffmann-La Roche, Monza, IT, Italy), β-PEA (β-phenylethylamine), L-NAME, 5-HT (Serotonin), methysergide (Sigma-Aldrich, Milano, IT, Italy). All the drugs were dissolved in distilled water. Klamin^®^ extract was dissolved in 0.5 mL water, and then sonicated (twice for 60 s) immediately prior to add to the bath. Chemicals were prepared as stock solution, which were diluted with Krebs solution on the experiment day.

### 2.5. Data and Statistical Analysis

The effects of Klamin^®^ were evaluated by measuring the mean amplitude of spontaneous contractions prior to and following drugs administration. The results are expressed as the changes in mean amplitude of the phasic contractions and reported as percentages of the values obtained in the control (e.g., 100% corresponds to the abolition of spontaneous activity). The concentration–response curve for each agonist was computer fitted using non-linear regression and the EC50 was calculated (GraphPad Prism, Version 4.01, GraphPad Software Inc., La Jolla, CA, USA).

All data are expressed as mean values ± standard error of the mean (S.E.M.). The letter *n* indicates the number of human colonic samples. Statistical analysis was performed by means of Student’s *t*-test or 2-way ANOVA followed by Bonferroni post-hoc test, when appropriate. A difference of median values with a *p*-value (*p*) < 0.05 was considered significant.

## 3. Results

### 3.1. Functional Study

**Figure 1:**
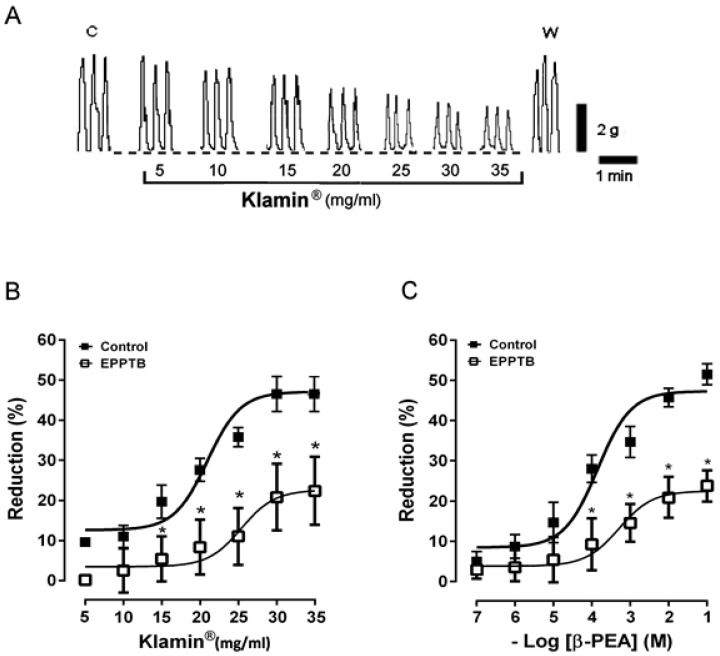
(**A**) Typical recordings showing the effects of increasing doses of Klamin^®^ (5–35 mg/mL) on the spontaneous contractions of human colon circular muscle. C = spontaneous contractions in control conditions. W = spontaneous contractions after washing out. Dotted line indicates the basal tone of the preparation. (**B**) Dose-response curves showing the effects of Klamin^®^ in the presence or in the absence of EPPTB (50 μM) (**C**) Concentration-response curves for the effects induced by β-PEA (0.1–100 μM) in the presence or in the absence of EPPTB (50 μM). Data are means ± S.E.M. (*n* = 6) and are expressed as percentage of reduction of the spontaneous contraction amplitude. * *p* ≤ 0.05 compared with the respective control conditions. On the contrary, as shown in Figure 2, increasing doses of AphaMax^®^ (5–35 mg/mL) only slightly reduced the amplitude of colonic spontaneous contractions (EC_50_ 0.9 mM; Cls 47 μM–0.1 mM) (Figure 2A,B).

Circular muscle strips of human colon exhibited spontaneous mechanical activity consisting of phasic contractions at a frequency of 4 ± 0.2 contractions per minute and amplitude of 3 ± 0.7 g (*n* = 30). Klamin^®^ (5–35 mg/mL) produced a dose-dependent decrease in the amplitude of the spontaneous contractions, without affecting the frequency or the basal tone (Figure 1A). These responses were reversible after washing out (Figure 1A). The maximal response (about 52% ± 6 of reduction of the spontaneous contractions) was obtained at 30 mg/mL Klamin^®^ (EC_50_ 0.24 μM; Cls 77–0.7 μM) (Figure 1B). The pre-treatment of the preparations with EPPTB (50 μM), a trace-amine receptor (TAAR1) antagonist, which per se did not modify the spontaneous mechanical activity, significantly reduced the Klamin^®^-induced effects (EC_50_ = 1 mM, Cls = 0.34–4 μM) (Figure 1B), supporting the hypothesis that β-PEA contained in AFA extract was responsible of the observed inhibitory effect. Then, the circular smooth muscle strips were exposed to cumulative increasing concentrations of synthetic β-PEA (0.1–100 μM). As shown by Figure 1C, β-PEA reduced the amplitude of the spontaneous contractions in a concentration-dependent manner. These reductions were of the same degree as that produced by Klamin^®^. Furthermore, the β-PEA effects were significantly antagonized by EPPTB (50 μM) (Figure 1C).

**Figure 3:**
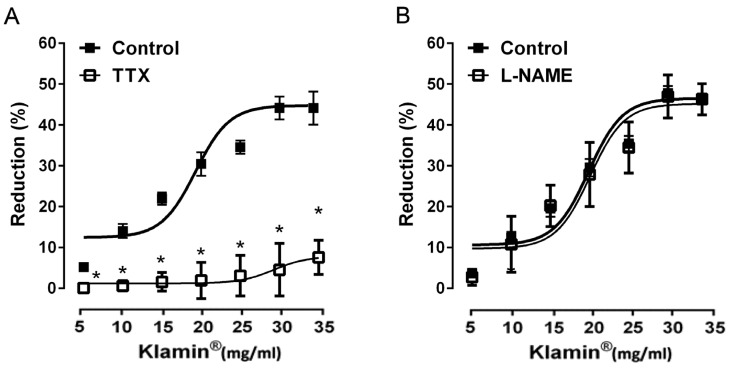
(**A**) Dose-response curves showing the effects of Klamin^®^ (5–35 mg/mL) before and after tetrodotoxin (1 μM) and (**B**) Dose-response curves showing the effects of Klamin^®^ (5–35 mg/mL) before and after Nω-nitro-L-arginine methyl ester (300 µM). Data are means S.E.M. (*n* = 6) and are expressed as percentage of reduction of the spontaneous contraction amplitude. * *p* ≤ 0.05 compared with the respective control conditions.

In order to investigate the mechanism of action underlying the observed effect, the responses of Klamin^®^ were tested in the presence of different drugs. The pre-treatment of smooth muscle strips with TTX (1 μM), a blocker of neural voltage-dependent Na+ channels, per se did not modify the spontaneous contractions indicating its balanced effect on excitatory and inhibitory nerves; on the contrary it abolished the contractility reduction to Klamin^®^ at all concentrations tested (Figure 3A). Klamin^®^-induced effects were not affected by L-NAME (300 μM), a nitric oxide synthase inhibitor, ruling out the involvement of nitric oxide (NO) (Figure 3B).

**Figure 4:**
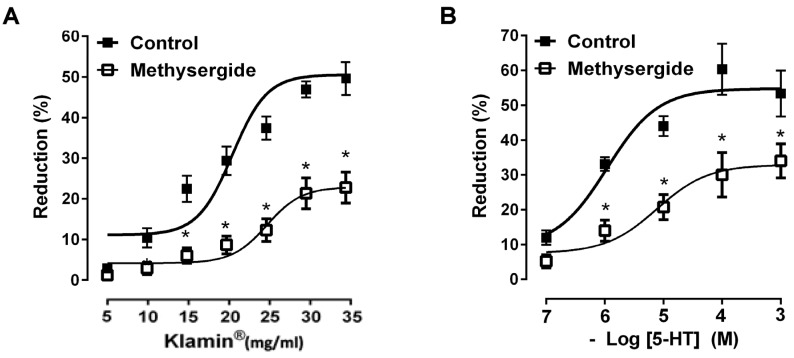
(**A**) Dose-response curves showing the effects of Klamin^®^ (5–35 mg/mL) in the absence and in the presence of a methysergide (10 μM). (**B**) Dose-response curves showing the effects of 5-HT (0.1–1 μM) before and after methysergide (10 μM). Data are means S.E.M. (*n* = 6) and are expressed as percentage of reduction of the spontaneous contraction amplitude. * *p* ≤ 0.05 compared with the respective control conditions.

In order to investigate if serotonin neurons were involved in the colon mechanical responses to Klamin^®^, we tested the Klamin^®^ -induced effects in the presence of methysergide (10 μM), a non-selective serotonin receptor antagonist. As shown in Figure 4A, the mechanical response were significantly antagonized by methysergide (10 μM), which per se failed to affect spontaneous contractions. The pre-treatment with methysergide (10 μM) was also able to significantly reduce (Figure 4B) the concentration-dependent mechanical effects induced by 5-HT (0.1–1 μM), consisting in a reduction of the amplitude of spontaneous contractions.

### 3.2. TAAR1 Expression Analysis

**Figure 5:**
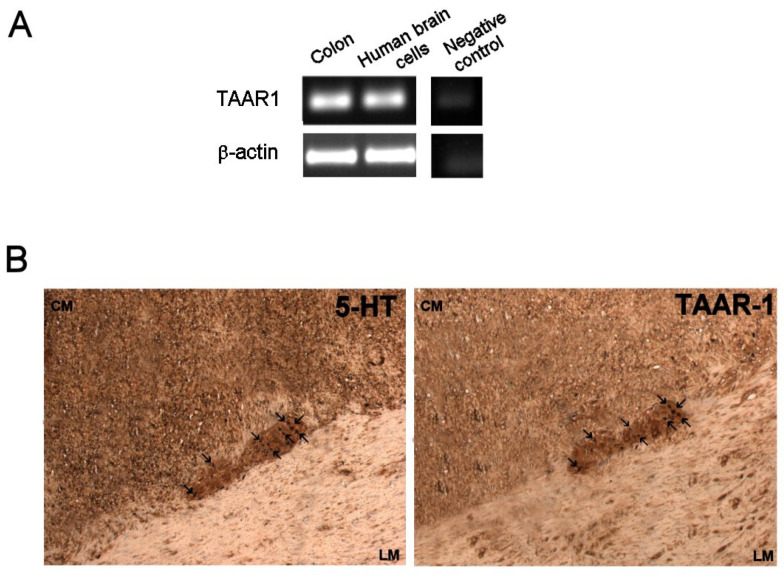
(**A**) Representative images of the reverse transcriptase-polymerase chain reaction (RT-PCR) results. A product of 297 bp corresponding to TAAR1 was detected in human colon and in human brain cells used as positive control. The expression of β-actin (396 bp) was used as loading control. Negative control was obtained without addition of cDNA. (**B**) Immunostaining of serotonin and TAAR1 in consecutive sections of the human colon. Left panel: immunostaining with antibody against serotonin. Right panel: immunostaining with antibody against TAAR1. The arrows indicate cells that co-expressed serotonin and TAAR1, as judged by their coincident position in equivalent area of adjacent sections. Original magnification: ×200.

RT-PCR analysis showed the presence of a 297 bp product relative to TAAR1 mRNA expression in human colon (Figure 5A). Moreover, immunohistochemical analysis revealed 5-HT-positive neurons overlapped with TARR1 positive neurons in consecutive sections of human colon, suggesting that TAAR1 are expressed in the myenteric plexus of human colon and that they are co-expressed with 5-HT (Figure 5B).

## 4. Discussion

The present results showed that Klamin^®^, a patented AFA extract, is able to reduce human colon contractility. The spasmolytic effect is due likely to β-PEA that is strongly concentrated in Klamin^®^. β-PEA, by binding to TAAR1, would induce 5-HT release from the enteric neurons, responsible for relaxant effects in colon circular muscle.

Klamin^®^ is a commercialized extract of the blue-green algae, AFA, containing high amount of beneficial nutrients, such as carotenoids, phycocyanins, and chlorophyll. These constituents have been suggested to be responsible for antioxidant, anti-inflammatory, anticancer and neuroprotective actions of AFA extract supplementation [23,33,34,35,36,37,38]. Klamin^®^ contains also elevated concentration of β-PEA, a natural neuromodulator [39], whose reduction in the CNS can lead to depression and affective disturbances [13,40]. The high content in β-PEA (20 mg/g) may explain the efficacy of Klamin^®^ supplementation in counteracting depression, anxiety, and other neuropathologies [24,25,26].

β-PEA has been demonstrated to act also in peripheral tissues such as vessel or gut smooth muscle [16,41,42,43]. Nevertheless, so far, the role of β-PEA as a neuromodulator of the ENS has not been reported yet. Both contractile and relaxant responses induced by this amine trace have been reported, depending on the gut region examined or animal species considered [16,17]. In this study, we used the organ bath, an experimental set-up, that allows us to analyze the mechanical activity of intestinal smooth, without extrinsic, neural or hormonal influence, in order to characterize the effects induced by Klamin^®^ on human colon contractility.

For the first time, our results revealed that Klamin^®^ reduced the amplitude of the spontaneous contractions of human circular smooth muscle in a dose-dependent manner, at least in old subjects, suggesting that Klamin^®^ can be considered as a spasmolytic agent. The observation that the response of the human colon to Klamin^®^ was significantly antagonized by EPTTB, a TAAR1 receptor antagonist [44], led us to hypothesize that β-PEA was responsible for the Klamin^®^ effects. In fact, TAAR1, a G protein–coupled receptor, belonging to the family of TAAR receptors, can be activated by the trace amines, including β-PEA [45]. In order to confirm our hypothesis, we examined the effects induced by exogenous β-PEA. Indeed, exogenous β-PEA reduced the colon spontaneous contractions in a concentration range similar to that contained in Klamin^®^ and previously shown to be efficacious in other experimental preparations [16,42,46] suggesting for the first time a role as negative modulator of human colon contractility. β-PEA exerted its effect with the same efficacy of Klamin^®^ and the response was significantly decreased in the presence of the TAAR1 antagonist, supporting the hypothesis about its involvement in the Klamin^®^-induced effect. On the other hand, AfaMax^®^, containing a lesser amount of β-PEA, slightly reduced the spontaneous contractions, strengthening our supposition.

In our study, β-PEA and Klamin^®^ were applied directly on the intestinal muscle strips, so their activity was similar. However, if considering oral intake, pure β-PEA would rapidly be degraded by selective MAO-B enzymes, highly expressed in muscular layer and myenteric plexus of human colon [47]. On the contrary, Klamin^®^, besides its natural β-PEA content, also contains molecules, such as AFA-phycocyanins, Mycosporine-like aminoacids (MAAs), and phycochrome, that have shown to be the most powerful natural selective MAO-B enzymes inhibitors [36]. This allows us to consider that Klamin^®^ could also exert its spasmolitic activity as an oral supplement.

It is interesting to note that, although humans and rodents demonstrate a high expression of TAARs in peripheral tissues including the gut [16,21,48], our study provides evidence, for the first time, of the presence of TAAR1 in human distal colon, as shown by the results of RT-PCR analysis and immunohistochemical experiments.

Furthermore, we examined if Klamin^®^ induced relaxant effects via a direct action on the smooth muscle cells and/or via an indirect action involving neural pathways. Indeed, mouse and human studies reported that TAAR1 receptors are located in intestinal epithelial cells as well as enteric neurons of both submucosa and myenteric plexus [22,49,50].

Our results showed that in the human colon, the Klamin^®^-induced response was abolished by TTX, a blocker of the voltage-dependent sodium channel responsible for the genesis and the propagation of neural action potential, suggesting that neurons within the ENS mediate the inhibitory effect of Klamin^®^ in human colon. Because of nitric oxide (NO) is the main inhibitory neurotransmitters in the gastrointestinal tract, including human colon [51], we verified its possible involvement in the Klamin^®^-induced effects. In our preparation, the blockade of NO-synthase by L-NAME did not affect the effects induced by AFA extract, ruling out the involvement of NO.

Because β-PEA has been shown to interact with serotoninergic neurons, leading to 5-HT release [17,52], we verified a possible involvement of 5-HT in the Klamin^®^-induced effects. The observation that methysergide significantly reduced the responses to Klamin^®^, at a concentration efficacious in antagonizing relaxant effects induced by 5-HT, suggested the involvement of 5-HT in the Klamin^®^ responses.

5-HT has been shown to mediate different effects on the gastrointestinal motility, depending on the considered segment and activated receptor sub type [46,53,54,55], although it is generally accepted that in the human small intestine 5-HT is responsible of contractions. On the contrary the predominant response in human colon is relaxation and the inhibition of spontaneous contractions, responses mainly attributed to activation of 5-HT4 and/or 5-HT7 receptors [56,57]. In our preparation, increasing concentrations of exogenous 5-HT induced a significant reduction of the amplitude of spontaneous contractions suggesting that serotonin is responsible of the contractility reduction of the human distal colon. Moreover, it is known that the serotonin source in the intestine could be neuronal, or it can be also located in other neurons outside the myenteric plexus, in enterochromaffin cells, or derived from systemic circulation.

Nevertheless, in our preparations, IHC results showed co-localization of 5-HT and TAAR1 in myenteric neurons, supporting the conclusion that Klamin^®^ reduces human colon contractility through the release of serotonin from myenteric neurons.

## 5. Conclusions

The results of the present study demonstrate that the Klamin^®^ is able to reduce the spontaneous contractions of the human colon. Likely, the relaxant effects are due to the β-PEA, that, by activating neural TAAR1, induces serotonin release from serotoninergic neurons of the myenteric plexus. The spasmolytic action of Klamin^®^ could be useful to counteract intestinal motility discomfort.

## Figures and Tables

**Figure 2:**
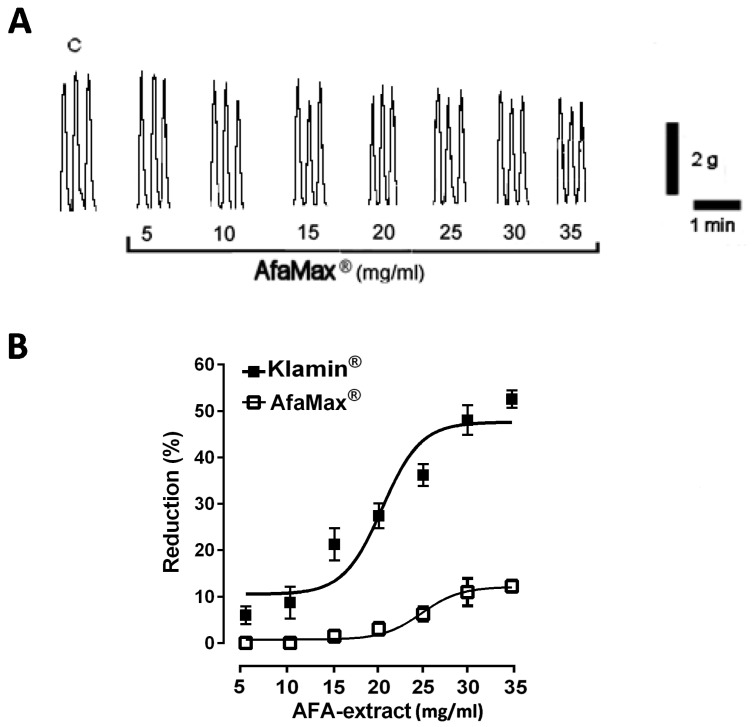
(**A**) Typical recordings showing the effects of increasing doses of AfaMax^®^ (5–35 mg/mL) on the spontaneous contractions of human colon circular muscle. C = spontaneous contractions in control conditions. (**B**) Dose-response curves showing the effects of the two AFA-extracts, Klamin^®^ (5–35 mg/mL) and AfaMax^®^ (5–35 mg/mL), on the spontaneous contractions of human colon circular muscle. Data are means S.E.M. (*n* = 6) and are expressed as percentage of reduction of the spontaneous contraction amplitude.
